# Biocompatible 2D Material Inks Enabled by Supramolecular Chemistry: From Synthesis to Applications

**DOI:** 10.1021/acs.accounts.4c00596

**Published:** 2025-01-08

**Authors:** Khaled Parvez, Cinzia Casiraghi

**Affiliations:** Department of Chemistry, The University of Manchester, Manchester M13 9PL, United Kingdom

## Abstract

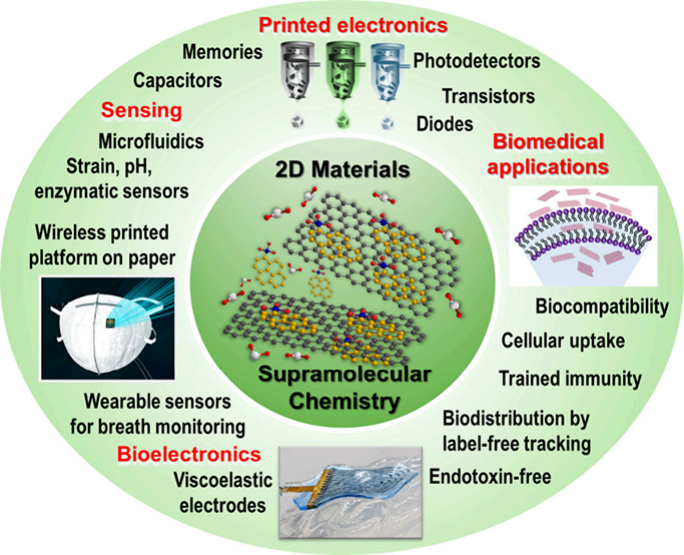

The emergence of two-dimensional (2D) materials, such as graphene, transition-metal dichalcogenides (TMDs), and hexagonal boron nitride (h-BN), has sparked significant interest due to their unique physicochemical, optical, electrical, and mechanical properties. Furthermore, their atomically thin nature enables mechanical flexibility, high sensitivity, and simple integration onto flexible substrates, such as paper and plastic.

The surface chemistry of a nanomaterial determines many of its properties, such as its chemical and catalytic activity. The electronic properties can also be modified by surface chemistry through changes in charge transfer or by the presence of surface states. Surface defects and functional groups can act as trap sites for excitons, hence affecting the optical properties. Furthermore, surface chemistry determines the stability and dispersibility of nanomaterials in colloidal dispersions as well as their biocompatibility and toxicity. In addition, the surface chemistry dictates how nanomaterials interact with biological systems, influencing cellular uptake, immune response, and biodistribution, to name a few examples. It is, therefore, crucial to be able to produce 2D materials with tunable surface chemistry to match target applications.

Because of their dimensionality, 2D materials can be easily functionalized with noncovalent and covalent approaches. This review delves into the role of supramolecular chemistry, which is based on noncovalent interactions, in achieving stable and highly concentrated water-based dispersions of 2D materials with specific surface chemistry.

In particular, we provide an overview of the recent progress made by our group in the field of solution-processed 2D materials produced by liquid-phase exfoliation with pyrene derivatives used as supramolecular receptors. We highlight the relationship between the structure of the pyrene derivative stabilizer and the concentration, stability, and lateral size and thickness distributions of the produced nanosheets. Subsequently, we give a short overview of the applications enabled by the supramolecular approach in printed electronics, sensing, bioelectronics, and in the biomedical field. We show that the careful design of the pyrene derivative enables us to achieve excellent stability of the material in the cellular medium, which is essential to accurately assess biological effects. We also highlight seminal case studies on the use of cationic graphene in the therapeutics of lysosomal storage disorders, and on the use of TMD nanosheets for trained immunity and as immune-compatible nanoplatforms, traceable at the single-cell and tissue (suborgan) levels.

This Account aims to provide a comprehensive guide for readers on the potential of the supramolecular approach for the design of 2D material dispersions with tailored surface chemistry. This approach is expected to be extremely attractive for many applications, from tissue engineering to energy storage devices, so we hope that this Account will drive further efforts and advancements in this field by ultimately leading to the integration of solution-processed 2D materials made by supramolecular chemistry into practical applications.

## Key References

- McManusD., VranicS., WithersF., Sanchez-RomagueraV., MacucciM., YangH., Water-based and biocompatible 2D crystal inks for all-inkjet-printed heterostructures. Nat. Nanotechnol.2017, 12, 343–35028135260 10.1038/nnano.2016.281.^1^*The work demonstrates a general approach to achieve concentrated and inkjet printable water-based 2D crystal formulations for fully printed electronic devices and includes the dose-escalation cytotoxicity assays of the inks using human lung and skin cell cultures*.
- ChenL., HuK., LuM., ChenZ., ChenX., ZhouT., Wearable Sensors for Breath Monitoring Based on Water-Based Hexagonal Boron Nitride Inks Made with Supramolecular Functionalization. Adv. Mater.. 2024, 36, 231262110.1002/adma.20231262138168037.^2^*This work demonstrates that the noncovalent functionalization of h-BN nanosheets with pyrene derivatives enables the use of the material as a sensing element in impedance-based sensors for breath monitoring*.
- ShinY., VranicS., Just-BaringoX., GaliSM, KisbyT., ChenY., Stable, concentrated, biocompatible, and defect-free graphene dispersions with positive charge. Nanoscale2020, 12, 12383–1239432490468 10.1039/d0nr02689a.^3^*This work demonstrates the synthesis and use of a family of cationic pyrene derivatives to produce biocompatible cationic graphene inks*.
- TringidesCM, VachicourasN., de LázaroI., WangH., TrouilletA., SeoBR, Viscoelastic surface electrode arrays to interface with viscoelastic tissues. Nat. Nanotechnol.2021, 16, 1019–102934140673 10.1038/s41565-021-00926-zPMC9233755.^4^*This work demonstrates a viscoelastic microelectrode array for soft biological tissue produced by adding graphene and carbon nanotubes into a hydrogel, enabling the device to adapt to changes in tissue conformation, while maintaining contact over time and without damage*.

## 1 Introduction

Two-dimensional (2D) materials are crystals that have lateral size much larger than their thickness; hence, they are usually produced as nanosheets with maximum thickness on the order of few nanometers.^5^ The low dimensionality of 2D materials gives rise to unique properties, arising from quantum confinement effects and their large surface area. Graphene is the most famous 2D material due to its combination of distinct properties, such as strength, flexibility, transparency, and outstanding electrical and thermal conductivity.^6^ The family of 2D materials includes additional crystals with properties complementary to those of graphene, such as h-BN, 2D TMDs, 2D transition-metal carbides and nitrides, and many others. Furthermore, 2D materials can be easily produced in solution, hence enabling use of simple, low cost, and mass scalable techniques for materials processing and device fabrication. Liquid phase exfoliation (LPE),^7,8^ one of the most used chemical exfoliation approaches, relies on the use of organic solvents for production of concentrated and stable 2D material dispersions.^7^ However, the high cost, high boiling point, and toxicity of organic solvents limit the use of 2D material inks in practical applications. Water is the most suitable solvent for many applications, and it is also mandatory for biomedical applications. However, many layered materials are insoluble or have a very low solubility in water.

This Account discusses the production of water-based 2D material dispersions obtained by using pyrene derivatives as supramolecular receptors, showing that this simple one-pot approach allows one to obtain highly stable graphene dispersions with tunable surface properties, hence enabling modulation of the cellular uptake and cytotoxicity of the material and the production of 2D materials with surface properties, tailored for specific applications simply by selecting the pyrene derivative structure.

## 2 Liquid-Phase Exfoliation in Water via Supramolecular Chemistry

The supramolecular approach is based on the formation of assemblies using noncovalent interactions. In this framework, a stabilizer (or exfoliating agent) is added to water and used to exfoliate 2D materials in water.^9−11^ Amphiphilic molecules, such as surfactants, polymers, and aromatic molecules, are effective stabilizers because they can noncovalently functionalize pristine graphene by surface adsorption, micelle formation, and/or π–π interaction (i.e., they share the electrons of π-orbitals in a noncovalent bond), leading to steric and/or electrostatic stabilization in water. While in principle any amphiphilic molecule can be used for LPE of 2D materials in water, the specific interactions between the stabilizer and the nanosheets and the water molecules determine not only the stability of the dispersion but also how much material can be exfoliated. Hence, it is crucial to identify the type of stabilizer that provides the lowest ratio of graphene to stabilizer concentration, because high concentrations of active material are usually needed for many applications. Furthermore, an excess of stabilizer can severely affect many properties of the material, including their biocompatibility (Section 3.4).^12^

### 2.1 Stability and Graphene Concentration

Polyaromatic hydrocarbons have been shown to be promising exfoliation agents for high-yield exfoliation of graphite, due to their effective adsorption onto the surface of graphene through π–π interactions. Pyrene derivatives, in particular, have been extensively investigated^12−18^ because a comparative study^14^ between traditional surfactants, polymers, and different types of pyrene derivatives demonstrated that the yield of exfoliated graphene with pyrene derivatives is the highest, compared to the one obtained with other types of stabilizers under the same experimental conditions. Furthermore, the exfoliation yield strongly depends on the amount of pyrene derivative used:^14^ at high stabilizer concentrations, the degree of dissociation in functional groups drops considerably. This alters the electrostatic charge distribution in the solution, badly affecting the adsorption of the stabilizer onto graphene. Also, the adsorbed molecules cannot induce strong repulsive forces between graphene sheets, by badly affecting the dispersions stability. It was noted^14^ that these pyrene derivatives are unlikely to form large structures in the solution or participate in self π–π stacking at higher concentrations, since the presence of functional groups at short distances from the pyrene basal plane sterically hinders such molecular arrangements. Furthermore, the graphene to stabilizer ratio in the final dispersion strongly depends on the specific structure of the pyrene derivative:^14^ it was suggested that the more electronegative functional groups in pyrene, e.g., a sulfonyl group, should provide better exfoliation efficiency by decreasing the electron density on the pyrene basal plane and thus increasing the affinity of the pyrene core with the surface of graphene by accepting electrons. Although similar conclusions were achieved in other works,^15^ detailed insights on the exfoliation were limited by the use of commercially available pyrene derivatives.

**Figure 1:**
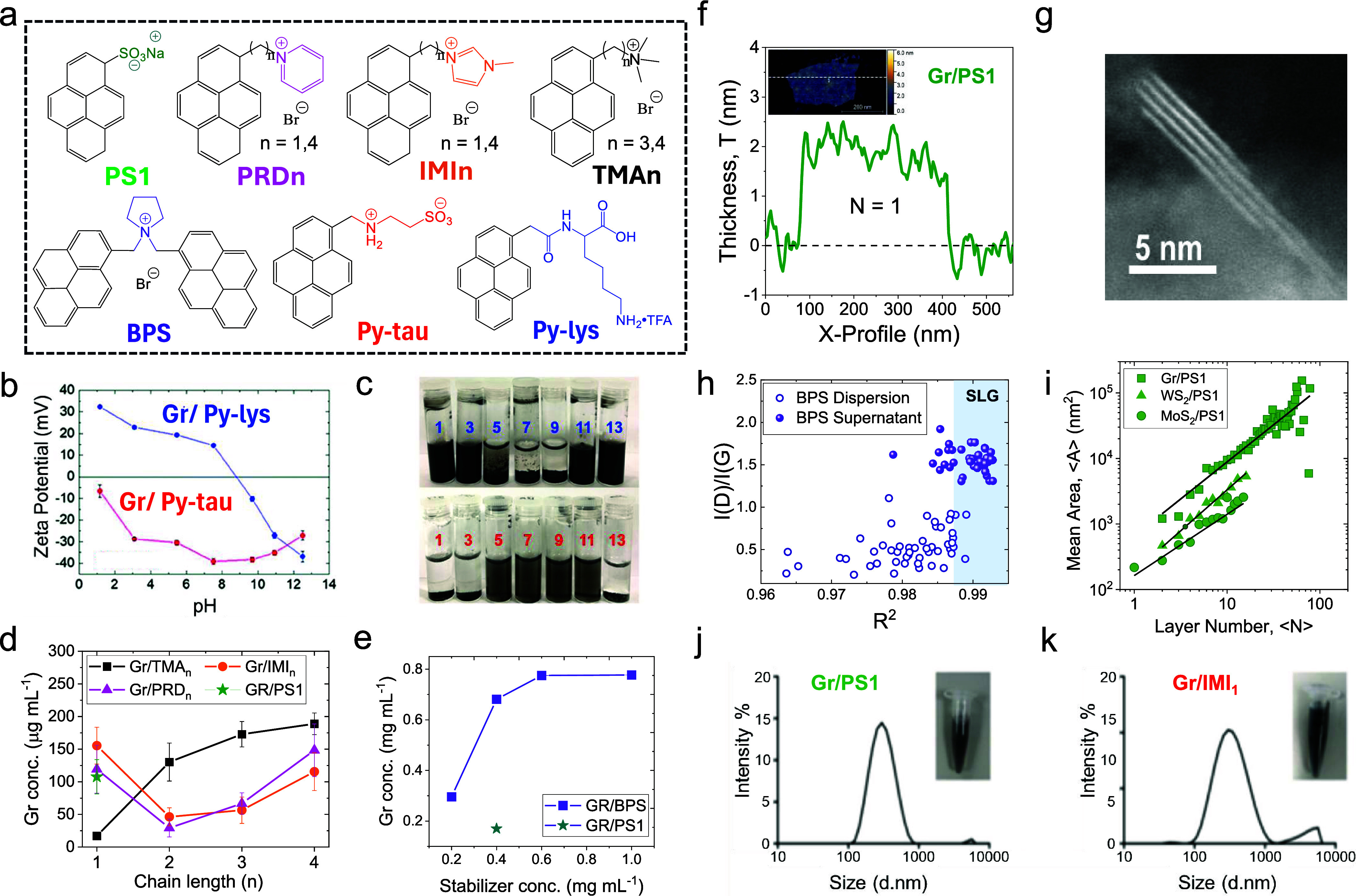
(a) Chemical structures of the pyrene derivatives discussed in this Account. (b) Zeta potential of graphene dispersions prepared by using py-lys (blue) and py-tau (red) and (c) corresponding photographs of the graphene dispersions obtained at different pH 1–13. (d) Concentration of graphene obtained by LPE in water with cationic pyrene derivatives as compared to PS1. (e) Concentration of graphene as a function of the concentration of BPS (purple) and PS1 (green). (f) Line profile extracted from the the AFM image of a graphene flake (inset) obtained by LPE with PS1. (g) Representative STEM image of a WS_2_ nanosheet obtained by LPE with PS1. (h) I(D)/I(G) vs residual fitting coefficient of the 2D peak from Raman measurements taken on individual graphene nanosheets obtained by LPE with BPS and extracted from the final dispersion and from the supernatant. (i) Average area versus average layer number of graphene, MoS_2_ and WS_2_ nanosheets obtained by PS1-assisted LPE. (j, k) Photographs (inset) and DLS measurements of washed graphene dispersions produced by LPE with PS1 and IMI_1_, respectively, after dispersion in the cell culture medium. [Panels (b) and (c) were reproduced with permission from ref (18). Copyright 2019, Royal Society of Chemistry, London. Panels (d), (h), (j), and (k) were reproduced from ref (3). Available under a CC-BY 3.0 license. Copyright 2020, Royal Society of Chemistry, London. Panel (e) was reproduced from ref (17). Available under a CC-BY 3.0 license. Copyright 2021, Royal Society of Chemistry, London. Panels (f) and (i) were reproduced with permission from ref (23). Copyright 2021, Elsevier, Ltd. Panel (g) reproduced from ref (16). Available under a CC-BY 3.0 license. Copyright 2014, IOP Publishing, Ltd.

Inspired by these seminal works, in 2013, our group reported the LPE of graphene in water using 1-pyrenesulfonic acid sodium salt (Py-1SO_3_, also known as PS1 (Figure 1a), which resulted in the production of highly crystalline flakes (i.e., free from C–O groups), high concentration with relatively good percentage of single layers and exceptional stability.^13^ We then successfully extended this method to the exfoliation of MoS_2_, MoTe_2_, MoSe_2_, WS_2_, and h-BN in water.^16^ By careful optimization of the sonication time and the centrifugation steps, we successfully prepared 2D material dispersions in water with concentration up to 10 mg/mL, while minimizing the amount of residual stabilizer,^1^ hence making the dispersions suitable for a wide range of applications (Section 3). Furthermore, our group demonstrated that the supramolecular approach can be used to successfully stabilize graphene produced by other exfoliation methods (e.g., anodic electrochemical exfoliation) in water.^19^

### 2.2 Tunable Surface Chemistry

In addition to the material concentration, another important parameter is the surface chemistry required by the target application, in particular, for sensing and biomedical applications. Indeed, the surface chemistry is expected to determine the sensor selectivity and to impact cellular internalization and other biological interactions.^20,21^ For example, cationic nanomaterials are typically preferred for biomedical applications (Section 3.4) because they show superior cellular internalization due their stronger affinity to the negatively charged lipid membrane, as compared to anionic nanomaterials.^22^ It is therefore important to be able to produce graphene nanosheets with tunable surface charge, e.g., cationic and anionic graphene dispersions (Gr^+^ and Gr^–^, respectively).

Our group demonstrated the production of Gr^+^ by using LPE in water assisted by various pyrene derivatives, all synthesized in house.^3,12,17,18,23,24^ We first investigated the use of amphoteric pyrene derivatives composed of a pyrene base linked to amino acids lysine (py-lys) or taurine (py-tau) (Figure 1a). The amphoteric functional group was selected because it enables the surface charge to change by tuning the pH. It was found that both amphoteric pyrene derivatives provide successful exfoliation of graphite; in particular, py-lys was able to provide good concentrations of stable Gr^+^ and Gr^–^ dispersions at low or high pH, respectively. In contrast, due to the very low p*K*_a_ value of the sulfonate group in py-tau, stable Gr^–^ were only observed for pH between 5 and 11 (Figures 1b and 1c).^18^

To further get insights on how to design the best pyrene derivative for production of Gr^+^, a family of cationic monopyrene derivatives with different type of functional groups (3-methylimidazolium = IMI_*n*_, pyridinium = PRD_*n*_, trimethylammonium = TMA_*n*_) and carbon linker length (*n* = 1–4) were synthesized (Figure 1a).^3^ Our results showed that, although the dispersions are all stable, the exfoliation efficiency, measured by the graphene concentration, strongly depends on the chain length and the type of functional group (Figure 1d). Molecular dynamics simulations indicated that a longer alkyl chain reduces steric hindrance of the functional group on the pyrene adsorption and increases thermodynamic stability of the solvation of polar groups, when the charge is localized. It was found that aromatic functional groups (e.g., IMI and PRD) enhance the adsorption of pyrene on graphene surface, but at the expense of lower dispersibility in water. Whereas the use of TMA functional group allowed better exfoliation efficiency with increased distance due to improved separation of adsorption of the pyrene base to the graphene surface and solubilization of localized change in water.

Since the affinity of the functional groups for the solvent medium is one of the key parameters to achieve stabilization in water, pyrene derivatives with relatively good aqueous solubility are typically selected for LPE. However, in 2020, we demonstrated that good solubility is not mandatory for a pyrene derivative to show efficient exfoliation:^17^ a bis-pyrene (BPS), functionalized with pyrrolidone central group (Figure 1a), was synthesized and used as a dispersant. Despite the BPS molecule being insoluble in water (up to the resolution of the spectrometer), it was found that the exfoliation efficiency is 3–5 times higher than that obtained with PS1 under the same conditions (Figure 1e). The enhanced exfoliation efficiency is attributed to the higher interaction strength between BPS and graphene than the interaction between BPS with water due to the strong π–π interactions provided by the two pyrene building blocks (which also cause BPS to be insoluble in water, further enhancing its adsorption onto graphene). The ability to produce highly concentrated dispersions allows for further fraction of the nanosheets by liquid cascade centrifugation,^25^ hence enabling production of stable and highly concentrated dispersions containing nanosheets with narrow lateral size distributions.^12^

These studies clearly demonstrated that the type of functional group, chain length, and size of the aromatic core strongly affect the exfoliation efficiency as they determine the efficiency of the molecule to get adsorbed onto graphene as well as the ability of the stabilizer to interact with the water molecules.

### 2.3 Material Characterization

Our group further investigated whether the chemical structure of the pyrene derivative affects the lateral size and thickness distributions of the exfoliated nanosheets by conducting a systematic atomic force microscopy (AFM) analysis on the nanosheets produced by LPE assisted with the pyrene derivatives. Table 1 reports the lateral size and thickness distributions of the nanosheets exfoliated with various pyrene derivatives. No matter the 2D material type, the smallest thickness measured was ∼1 nm (Figure 1f). Using this value as the effective thickness of a monolayer, the average *N*, ⟨*N*⟩, of the graphene nanosheets produced by pyrene derivatives is ∼7, while the ⟨*N*⟩ value of the MoS_2_ and WS_2_ nanosheets is ∼4. This is in good agreement with results obtained by transmission electron microscopy (TEM).^3,17,18,23^Figure 1h shows a representative high-resolution high angle annular dark field (HAADF) scanning transmission electron microscopy (STEM) image of a WS_2_ flake whose basal plane was oriented parallel to the imaging electron beam, hence enabling us to determine the number of layers directly from the number of parallel bright lines visible in the HAADF image. The image shows that the flake has a maximum thickness of 4 layers.^16^ The AFM analysis is also in good agreement with measurements obtained by Raman spectroscopy, which can only provide qualitative results for solution-processed 2D materials. In the case of solution-processed graphene, the Raman analysis is based on the shape of the 2D peak. This is fitted with a single Lorentzian line shape and used to distinguish between single-layer graphene (SLG), few-layer sheets (FLG) and graphitic material (>10 layers with AB stacking) by evaluating the 2D peak fit residual, *R^2^*. SLG is assigned to a single symmetric 2D peak with *R*^2^ > 0.987, FLG to a single asymmetric peak with *R*^2^ < 0.987, while the graphitic material is distinguished by its characteristic peak shape with shoulder. Figure 1h shows the intensity ratio between the D peak and the G peak, *I*(D)/*I*(G), as a function of *R*^2^ for a graphene dispersion and its supernatant obtained by LPE in water with BPS. The Raman analysis shows that the supernatant is composed of a higher number of SLG, compared to the dispersion.^17^

**Table 1:** Physicochemical Properties of Graphene Exfoliated with Various Pyrene Derivatives^a^

		Pyrene Derivative						
property	technique	PS1	TMA_*n*_	IMI_*n*_	PRD_*n*_	BPS	Py-lys	Py-tau
type of graphene		Gr^–^	Gr^+^	Gr^+^	Gr^+^	Gr^+^	Gr^+^ and Gr^–^	Gr^–^
average lateral size (nm)	AFM	221 ± 91	161 ± 92	165 ± 70	172 ± 85	159 ± 88	∼221	162 ± 62
average thickness (nm)	AFM	6.8 ± 2.4	5.7 ± 2.3	5.5 ± 1.8	5.7 ± 2.3	5.8 ± 3.7	2–10	7.2 ± 2.2
zeta potential (mV)	DLS	–36	39			∼40	32 (pH 1)	–30.4 (pH 4)
% of SLG	Raman spectroscopy		27.5%	40%	42.5%	∼58%	∼44%	∼42%
elemental composition	XPS^b^	C: 93.6%	C: 95.6%	–	–	–	–	–
		O: 4.9%	O: 3.8%					
		N: 0.7%	N: 0.3%					
		S: 0.8%	S: 0.2%					

a Data taken from refs (3), (12), (13), (18), and (23).

b XPS = X-ray photoelectron spectroscopy.

The AFM study conducted by our group also demonstrated a power-law scaling of nanosheet average area ⟨*A*⟩ vs ⟨*N*⟩ (Figure 1i), characteristic of each type of 2D material, but independent of the type of pyrene used.^23^ This is in agreement with previous works on 2D materials produced by LPE in organic solvents and water/surfactants solutions, demonstrating that this scaling law between lateral size and thickness is related only to the LPE process.^25,26^

The graphene dispersions obtained by LPE in water with PS1, PRD_*n*_, IMI_*n*_, TMA_*n*_ (*n* = 1–4) show excellent stability both in water and in the cellular medium.^3^Figures 1j and 1k shows the size distribution measured by dynamic light scattering (DLS) of the graphene dispersions obtained with PS1 and IMI_1_ that were first dispersed in cell culture medium (RPMI) supplemented with 10% fetal bovine serum (FBS) at 50 μg mL^–1^ and incubated for 24 h at 37 °C and 5% CO_2_, and then centrifuged, washed, and redispersed in water. The measurements show monodispersed size distribution and a lack of precipitation of material.^3^

## 3 Applications

### 3.1 Printed Electronics

**Figure 2:**
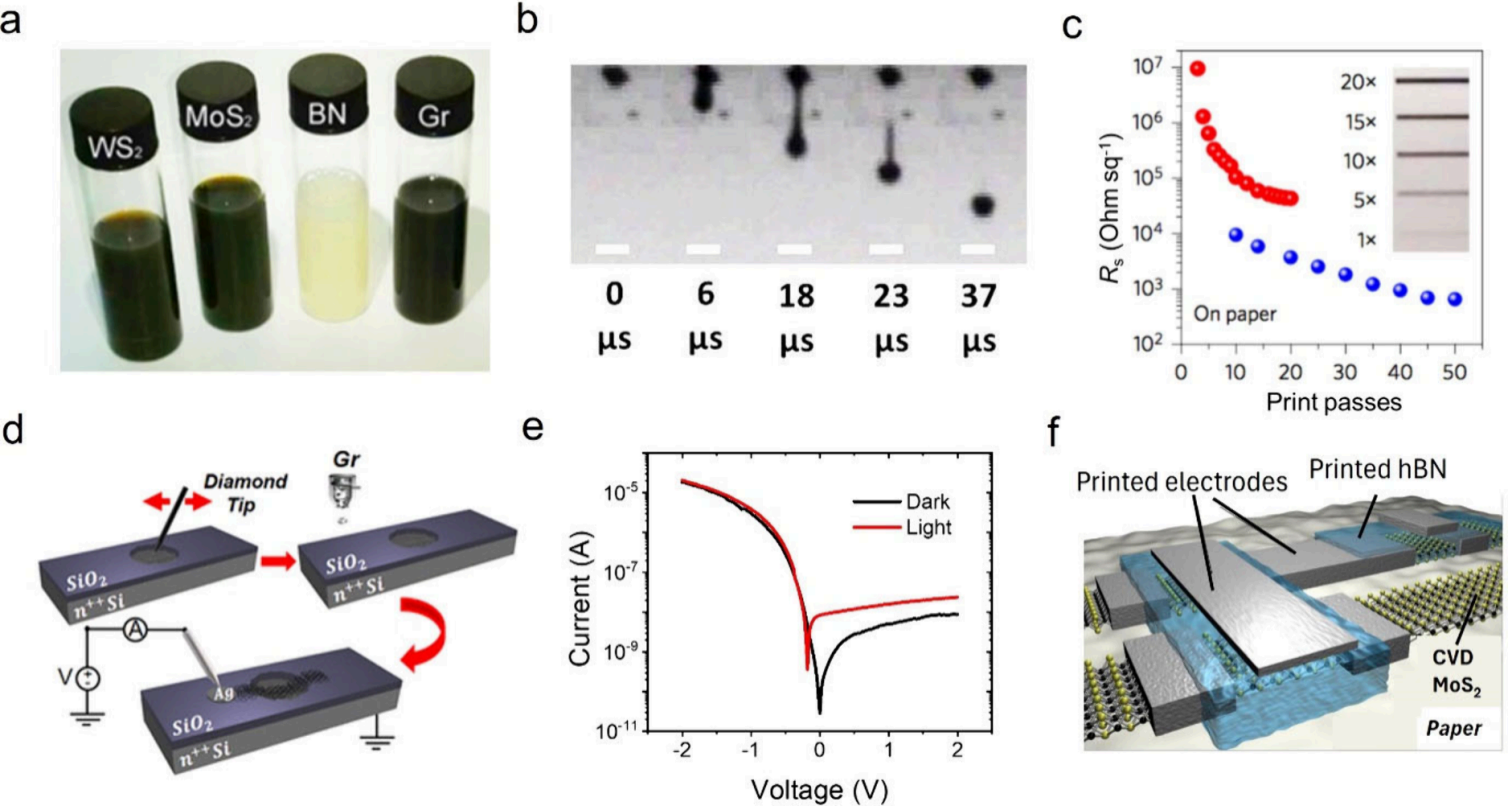
(a) Image of the water-based and printable 2D material inks. (b) Optical image of the graphene droplet formation vs time of the graphene ink after formulation engineering, showing filament tail formation and retracting into the main droplet, hence confirming printability. Scale bars = 50 μm. (c) Sheet resistance versus the number of printing passes of printed graphene lines on paper. Inset: optical image of the printed lines. (d) Schematic of the silicon-graphene diode fabrication and (e) its electrical characterization. (f) Schematic of a printed transistor on paper made by inkjet printing electrodes and dielectric onto CVD-grown MoS_2_. [Panels (a), (b), and (c) were reproduced with permission from ref (1). Copyright 2017, Macmillan Publishers, Ltd. Panels (d) and (e) were reproduced from ref (39). Copyright 2022, American Chemical Society, Washington, DC. Panel (f) was reproduced from ref (36). Available under a CC-BY 4.0 license. Copyright 2020, the Authors.]

The first demonstration of printed devices was made with graphene inks based on organic solvents,^27,28^ hence, opening issues on safety and sustainability. Furthermore, organic solvents have high boiling points; therefore, they are difficult to remove from the printed film. As discussed in Section 2, the supramolecular approach based on PS1 enables the production of stable and concentrated dispersions of 2D materials in water. However, water is not a suitable solvent for printing hydrophobic materials with a piezoelectric inkjet printer, which is commonly used in research laboratories. To achieve an inkjet printable formulation, the ink must have viscosity η, surface tension γ, and density ρ within certain ranges for a set nozzle diameter α. The inverse Ohnesorge number (*Z*) is commonly used to predict if an ink will form stable drops:^29^
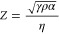
Graphene printable formulations in organic solvents have been achieved for Z ≈ 20.^27^ However, the *Z* value of water is ∼40 (using α = 21.5 μm). Our group demonstrated that water-based, stable, and printable 2D material-based inks (Figure 2a), can be obtained by first using the supramolecular LPE based on PS1 and then by performing careful formulation engineering to lower γ and increase η of water until Z is ∼20.^1^ For the fabrication of vertical devices, such as heterostructures, a binder was also added to the ink to minimize redispersion of different printed materials at the interface.^1^ Stable droplet formation of the water-based inks of 2D materials is observed after formulation engineering (Figure 2b), hence confirming the printability of the as-modified water-based inks. The inks can be printed on various substrates, from rigid to flexible. In particular, the porosity of the paper substrate enables to minimize the sheet resistance (*R*_s_) of the printed graphene films, compared to other substrates, under the same printing conditions (Figure 2c). Various devices, such as photodetectors,^1,30,31^ memories,^1,32^ transistors,^33−37^ capacitors,^38^ and diodes,^33,39,40^ have been demonstrated. Inkjet printing can also be easily integrated with other approaches, such as silicon-based technology^39^ and chemical vapor deposition (CVD)^32,34−36^ (see Figures 2d–f).

### 3.2 Sensing

**Figure 3:**
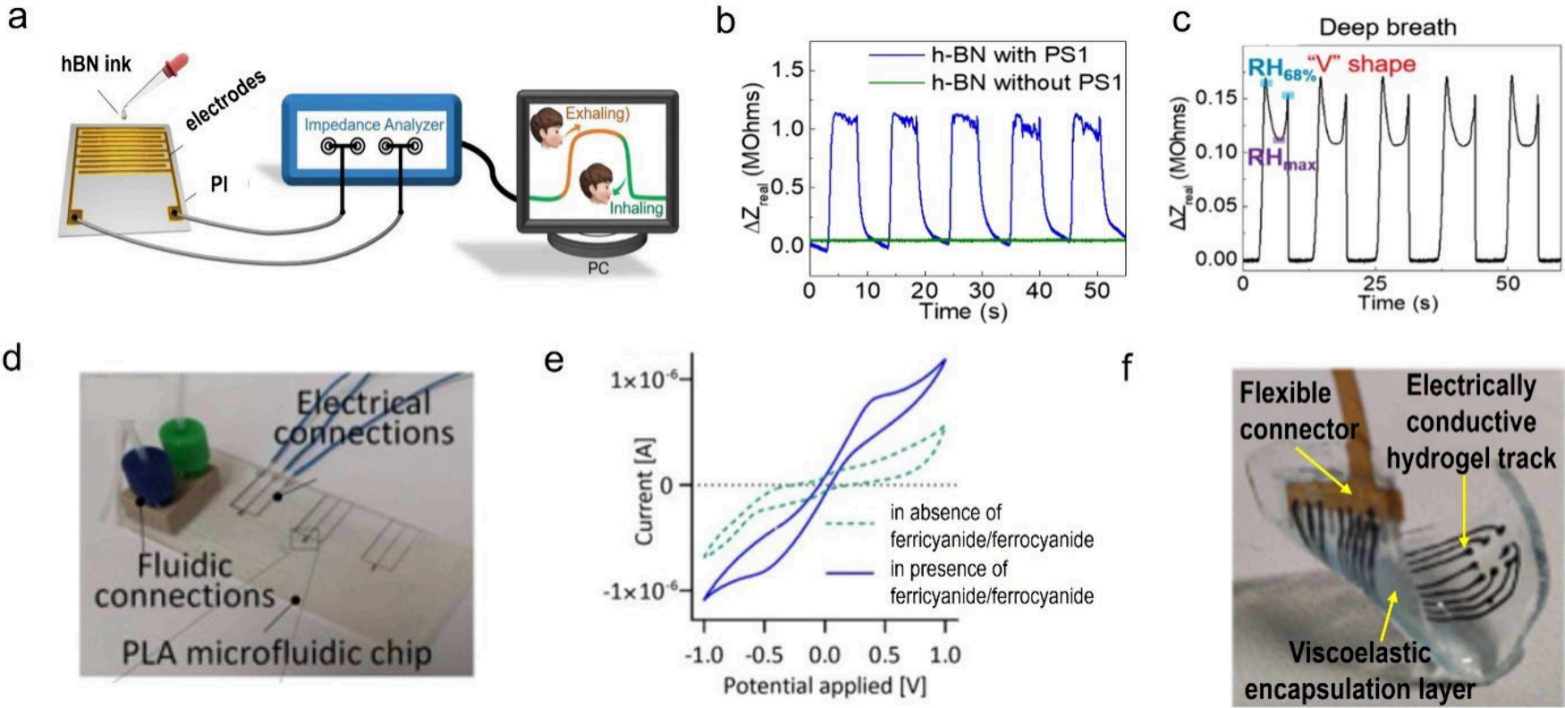
(a) Schematic of the hBN-based humidity sensor fabrication and testing. (b) Changes in the real part of impedance of the sensors made with hBN nanosheets with (blue line) and without PS1 (green line), by changing the relative humidity between 20% and 60% for five times. (c) Changes in the real part of the impedance during deep breathing. (d) Photograph of the prototype microfluidic device. (f) Electrochemical characterization of the microfluidic device. (g) Photograph of the fully viscoelastic array. [Panels (a)–(c) were reproduced with permission from ref (2). Available under a CC-BY 4.0 license. Copyright 2024, the Authors. Panels (d) and (e) were reproduced from ref (45). Copyright 2018, American Chemical Society, Washington, DC. Panel (f) was reproduced with permission from ref (4). Copyright 2021, Springer Nature, Ltd.]

2D materials are very attractive for the development of sensors, because their large surface area provides highest responsiveness, sensitivity, and reversibility, combined with the lowest limit of detection in the sensing process.^41,42^ As discussed in Section 2, their surface chemistry can be easily tuned by selecting (supra)molecular receptors that can recognize the target species via one or multiple noncovalent interactions.^41^ In this framework, the pyrene derivative, used as stabilizer during the LPE, provides an added value to the ink because it determines the material’s selectivity. This concept has been recently demonstrated by our group:^2^ a impedance-based humidity sensor was made with h-BN nanosheets obtained by LPE with PS1 (Figure 3a). The noncovalent functionalization with PS1 allows h-BN, which is hydrophobic, to be able to interact with water molecules via hydrogen bonding, hence producing changes in the device impedance when changing the relative humidity. The same device made with h-BN exfoliated in isopropanol does not show any sensitivity to the humidity, hence confirming that PS1 acts as a supramolecular receptor for the humidity sensor (Figure 3b). This device is particularly suitable for breath monitoring because the real part of the impedance shows a characteristic line shape over the whole breathing cycle (Figure 3c), hence enabling one to identify subtle changes in the exhaling and inhaling of the air by simply looking at changes of the signal from this characteristic line shape.^2^

Another promising approach in sensing and biosensing is based on the integration of sensors into microfluid devices.^43,44^ In a seminal work,^45^ a microelectrode array was fabricated by inkjet printing our graphene ink (Section 3.1) directly onto poly(lactic acid).^45^ The electrodes were integrated into a microfluidic channel (Figure 3d) and used to measure the redox currents of the ferricyanide/ferrocyanide redox reaction. A negative control in the absence of potassium ferricyanide/ferrocyanide (Figure 3e) showed no peaks, thereby confirming the suitability of the printed graphene electrodes for microfluidics.^45^

A variety of printed devices, such as photodetectors,^30^ enzymatic sensors,^46^ strain,^47^ and pH sensors^48^ have been demonstrated. Furthermore, the ability to print on paper (Section 3.1) allows the sensing platform to be easily connected to a printed near-field communication antenna, hence leading to a fully printed and wireless sensing platform on paper.^30,48^

### 3.3 Hydrogels for Bioelectronics

The supramolecular approach provides stable and highly concentrated dispersions of nonoxidized graphene in water (Section 2) that can be therefore used as conductive fillers for hydrogels for use in various applications. For example, a viscoelastic microelectrode array for soft biological tissues was made by adding a mixture of graphene produced by LPE with PS1 and carbon nanotubes to a hydrogel made from an ionically conductive alginate. The electrode array was encapsulated into a thin layer of an insulating physically entangled viscoelastic material covalently attached to a thicker alginate-based tough gel with similar viscoelasticity as the alginate-only gels, hence leading to the fabrication of a fully viscoelastic and conformable device (Figure 3f), which was tested in vitro and in vivo. The device is able to adapt to changes in tissue conformation, while maintaining contact over time without damaging the underlying mock tissue.^4^

### 3.4 Biomedical Applications

The most studied 2D material for biomedical applications is graphene oxide (GO), due to its hydrophilicity and chemical reactivity, provided by the presence of C–O groups. The safety profile and biological effects of GO have been extensively studied.^49^ However, the exploitation of GO for biomedical applications requires the production of highly purified and thin nanometer-sized graphene.^50^ The surface chemistry of GO is indeed strongly dependent on the production method,^51^ which also involves the use of strong chemical agents, rising issues of sustainability along the production value chain.^52^ Furthermore, GO is dispersable in water, but it does aggregate in solutions rich in salts or proteins such as cell medium and serum.^53^ It is therefore important to develop alternative methods for the production of water-based and biocompatible graphene dispersions. Our group used the supramolecular approach to produce graphene nanosheets with controlled morphology and tunable surface chemistry to get a deeper understanding of the relationship between the 2D material structure and biological effects, which is crucial for ensuring the effective and safe use of 2D materials in practical applications.^21^

**Figure 4:**
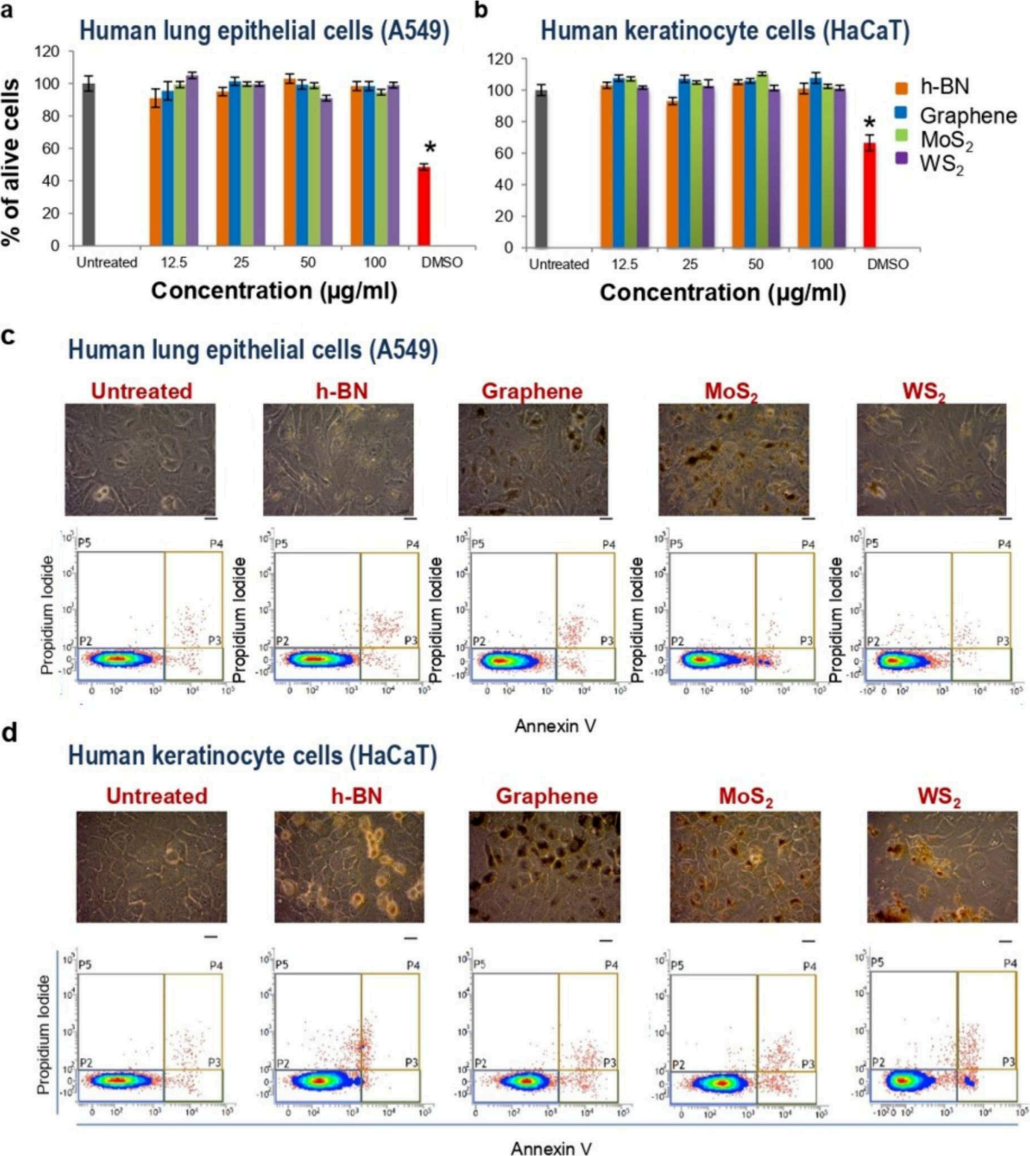
(a) Cell membrane damage in A549 and HaCaT cells assessed using the modified LDH assay when exposed to escalating doses of 2D material inks or 10% dimethyl sulfoxide (DMSO) for 24 h. (b, c) Top row: optical microscopy images of untreated and cells treated with 50 μg mL^–1^ of inks for 24 h. Scale bars = 10 μm. Bottom row: assessment of cytotoxicity of the material by flow cytometry after staining and incubation with cellular markers of apoptosis (annexin V) and necrosis (PI incubation of the material, stained with cellular markers of apoptosis (annexin V) and necrosis (PI), for 24 h. In the bivariate plots, live cells are represented in the P2 region, early apoptotic are represented in the P3 region, late apoptotic and/or necrotic cells are shown in P4 region and necrotic cells are in the P5 region. [Reproduced with permission from ref (1). Copyright 2017, Springer Nature, Ltd.]

Excellent stability of the material in the cellular medium is a necessary requirement to accurately access the material biocompatibility and cellular uptake. As demonstrated in Section 2, the supramolecular LPE based on pyrene derivatives provides 2D materials with exceptional stability in the medium, hence enabling us to conduct their safety assessments. These were made by in vitro studies, using cell culture models representative of the human tissues that constitute the primary physiological barriers in nonoccupational (i.e., skin exposure of consumers) and occupational (i.e., pulmonary exposure of workers in the production lines of such materials) scenarios.^1^ Overall, no significant morphological changes indicative of cell death were observed after exposing the material to human lung (human alveolar epithelial cells: A549) and skin (human keratinocytes: HaCaT) cells, up to the highest concentration tested (100 μg mL^–1^) (Figure 4).^1^ Our results show that different 2D materials produced by LPE with the PS1 stabilizer are all biocompatible, despite having different lateral size and thickness distributions. This suggests that biocompatibility is determined by the surface chemistry since the nanosheets are all functionalized with PS1, at least within the typical range of size and thickness of our nanosheets. This has been further confirmed by replacing PS1 with BPS: in this case, cytotoxicity tests using human lung epithelial cell line BEAS-2B showed reduced biocompatibility in comparison to graphene produced by PS1 only when using BPS concentrations above 0.2 mg mL^–1^, no matter the lateral size and thickness distributions of the nanosheets.^17^ Hence, the type and amount of pyrene derivative adsorbed on the nanosheets strongly determine its biocompatibility.

We also investigated the cellular uptake of graphene nanosheets obtained by LPE with PS_1_, IMI_1_, PRD_1_, and TMA_3_.^3^ Cellular uptake was observed to strongly depend on the surface charge: Gr^–^ is the least internalized by the cells, whereas all Gr^+^ are taken up more efficiently, although to a different extent after 24 h of treatment at the used dose of 50 μg mL^–1^. In the same study, it was noted that Gr^+^ always localizes in the peri-nuclear regions, suggesting lysosomal subcellular localization without causing toxicity. This is a very remarkable finding as alternative cationic nanomaterials have been shown to induce cell death via lysosomal or rupture of the plasma membrane.^54,55^ Following this observation, we have recently investigated the use of Gr^+^ produced by LPE with IMI_3_ as vector for improving the therapeutic approach of lysosomal storage disorders.^56^

While most of the safety essays have been performed on graphene nanosheets, biological profiles were recently performed on TMDs produced by LPE with PS1.^57^ Both MoS_2_ and WS_2_ were found to be endotoxin-free using the TNF-α expression test (TET) and no cytotoxicity was observed up to a dose of 100 μg mL^–1^, despite the strong uptake by primary human monocyte-derived macrophages (HMDMs) when exposed to the TMDs for 24 h.^57^ Furthermore, MoS_2_ was observed to trigger trained immunity in macrophages, and that Mo becomes bioavailable in macrophages that have taken up the MoS_2_ nanosheets.^57^ In a second study, in-depth cellular effects, as well as in vivo biodistribution of the TMDs were reported through the simultaneous label-free tracking of their immune cell interactions, both ex vivo in human peripheral blood mononuclear cells and in vivo in mice by single cell mass cytometry.^58^

## 4 Conclusions

This Account shows that the supramolecular approach allows the one-pot production of stable and highly concentrated 2D material dispersions in water with specific surface chemistry, suitable for various applications. The list of applications is expected to increase following further research aimed at achieving better understanding on the nanosheet-stabilizer interactions and the electrical and thermal transport of films made of random networks of nanosheets.^35,59−61^ In terms of biomedical applications, further research, for example, on biodistribution, translocation to secondary organs, accumulation, degradation, and clearance, is still needed to fully reveal the potential of 2D materials inks enabled by supramolecular chemistry.

Overall, water-based 2D materials produced by LPE with pyrene derivatives offer substantial potential and opportunities in several applications, from electronics to energy storage and management, and from bioelectronics to biomedical applications.

## References

[ref1] McManus D.; Vranic S.; Withers F.; Sanchez-Romaguera V.; Macucci M.; Yang H.; Sorrentino R.; Parvez K.; Son S.-K.; Iannaccone G.; et al. Water-based and biocompatible 2D crystal inks for all-inkjet-printed heterostructures. Nat. Nanotechnol. 2017, 12, 343–350. 10.1038/nnano.2016.281. 28135260

[ref2] Chen L.; Hu K.; Lu M.; Chen Z.; Chen X.; Zhou T.; Liu X.; Yin W.; Casiraghi C.; Song X. Wearable Sensors for Breath Monitoring Based on Water-Based Hexagonal Boron Nitride Inks Made with Supramolecular Functionalization. Adv. Mater. 2024, 36, 2312621 10.1002/adma.202312621. 38168037

[ref3] Shin Y.; Vranic S.; Just-Baringo X.; Gali S. M.; Kisby T.; Chen Y.; Gkoutzidou A.; Prestat E.; Beljonne D.; Larrosa I.; et al. Stable, concentrated, biocompatible, and defect-free graphene dispersions with positive charge. Nanoscale 2020, 12, 12383–12394. 10.1039/D0NR02689A. 32490468

[ref4] Tringides C. M.; Vachicouras N.; de Lázaro I.; Wang H.; Trouillet A.; Seo B. R.; Elosegui-Artola A.; Fallegger F.; Shin Y.; Casiraghi C.; et al. Viscoelastic surface electrode arrays to interface with viscoelastic tissues. Nat. Nanotechnol. 2021, 16, 1019–1029. 10.1038/s41565-021-00926-z. 34140673 PMC9233755

[ref5] Novoselov K. S.; Jiang D.; Schedin F.; Booth T. J.; Khotkevich V. V.; Morozov S. V.; Geim A. K. Two-dimensional atomic crystals. Proc. Natl. Acad. Sci. U. S. A. 2005, 102, 10451–10453. 10.1073/pnas.0502848102. 16027370 PMC1180777

[ref6] Novoselov K. S.; Fal’ko V. I.; Colombo L.; Gellert P. R.; Schwab M. G.; Kim K. A roadmap for graphene. Nature 2012, 490, 192–200. 10.1038/nature11458. 23060189

[ref7] Hernandez Y.; Nicolosi V.; Lotya M.; Blighe F. M.; Sun Z.; De S.; McGovern I. T.; Holland B.; Byrne M.; Gun’Ko Y. K.; et al. High-yield production of graphene by liquid-phase exfoliation of graphite. Nat. Nanotechnol. 2008, 3, 563–568. 10.1038/nnano.2008.215. 18772919

[ref8] Coleman J. N. Liquid Exfoliation of Defect-Free Graphene. Acc. Chem. Res. 2013, 46 (1), 14–22. 10.1021/ar300009f. 22433117

[ref9] Hu C.-X.; Shin Y.; Read O.; Casiraghi C. Dispersant-assisted liquid-phase exfoliation of 2D materials beyond graphene. Nanoscale 2021, 13, 460–484. 10.1039/D0NR05514J. 33404043

[ref10] He Y.; Andrade A. F.; Ménard-Moyon C.; Bianco A. Biocompatible 2D Materials via Liquid Phase Exfoliation. Adv. Mater. 2024, 36, 2310999 10.1002/adma.202310999. 38457626

[ref11] Ciesielski A.; Samori P. Graphene via sonication assisted liquid-phase exfoliation. Chem. Soc. Rev. 2014, 43, 381–398. 10.1039/C3CS60217F. 24002478

[ref12] Hu C.-X.; Read O.; Shin Y.; Chen Y.; Wang J.; Boyes M.; Zeng N.; Panigrahi A.; Kostarelos K.; Larrosa I.; et al. Effects of Lateral Size, Thickness, and Stabilizer Concentration on the Cytotoxicity of Defect-Free Graphene Nanosheets: Implications for Biological Applications. ACS Appl. Nano Mater. 2022, 5, 12626–12636. 10.1021/acsanm.2c02403. 36185165 PMC9513747

[ref13] Yang H.; Hernandez Y.; Schlierf A.; Felten A.; Eckmann A.; Johal S.; Louette P.; Pireaux J. J.; Feng X.; Mullen K.; et al. A simple method for graphene production based on exfoliation of graphite in water using 1-pyrenesulfonic acid sodium salt. Carbon 2013, 53, 357–365. 10.1016/j.carbon.2012.11.022.

[ref14] Parviz D.; Das S.; Ahmed H. S. T.; Irin F.; Bhattacharia S.; Green M. J. Dispersions of Non-Covalently Functionalized Graphene with Minimal Stabilizer. ACS Nano 2012, 6, 8857–8867. 10.1021/nn302784m. 23002781

[ref15] Schlierf A.; Yang H.; Gebremedhn E.; Treossi E.; Ortolani L.; Chen L.; Minoia A.; Morandi V.; Samorì P.; Casiraghi C.; et al. Nanoscale insight into the exfoliation mechanism of graphene with organic dyes: effect of charge, dipole and molecular structure. Nanoscale 2013, 5, 4205–4216. 10.1039/c3nr00258f. 23467481

[ref16] Yang H.; Withers F.; Gebremedhn E.; Lewis E.; Britnell L.; Felten A.; Palermo V.; Haigh S.; Beljonne D.; Casiraghi C. Dielectric nanosheets made by liquid-phase exfoliation in water and their use in graphene-based electronics. 2D Mater. 2014, 1, 011012 10.1088/2053-1583/1/1/011012.

[ref17] Shin Y.; Just-Baringo X.; Boyes M.; Panigrahi A.; Zarattini M.; Chen Y.; Liu X.; Morris G.; Prestat E.; Kostarelos K.; et al. Enhanced liquid phase exfoliation of graphene in water using an insoluble bis-pyrene stabiliser. Faraday Discuss. 2021, 227, 46–60. 10.1039/C9FD00114J. 33295354

[ref18] Shin Y.; Just-Baringo X.; Zarattini M.; Isherwood L. H.; Baidak A.; Kostarelos K.; Larrosa I.; Casiraghi C. Charge-tunable graphene dispersions in water made with amphoteric pyrene derivatives. Mol. Syst. Des. Eng. 2019, 4, 503–510. 10.1039/C9ME00024K.

[ref19] Parvez K.; Worsley R.; Alieva A.; Felten A.; Casiraghi C. Water-based and inkjet printable inks made by electrochemically exfoliated graphene. Carbon 2019, 149, 213–221. 10.1016/j.carbon.2019.04.047.

[ref20] Harper S.; Usenko C.; Hutchison J. E.; Maddux B. L. S.; Tanguay R. L. In vivo biodistribution and toxicity depends on nanomaterial composition, size, surface functionalisation and route of exposure. J. Exp. Nanosci. 2008, 3, 195–206. 10.1080/17458080802378953.

[ref21] Fadeel B.; Bussy C.; Merino S.; Vázquez E.; Flahaut E.; Mouchet F.; Evariste L.; Gauthier L.; Koivisto A. J.; Vogel U.; et al. Safety Assessment of Graphene-Based Materials: Focus on Human Health and the Environment. ACS Nano 2018, 12, 10582–10620. 10.1021/acsnano.8b04758. 30387986

[ref22] Chen L.; McCrate J. M.; Lee J. C. M.; Li H. The role of surface charge on the uptake and biocompatibility of hydroxyapatite nanoparticles with osteoblast cells. Nanotechnol. 2011, 22, 105708 10.1088/0957-4484/22/10/105708. PMC3144725 21289408

[ref23] Read O.; Shin Y.; Hu C.-x.; Zarattini M.; Boyes M.; Just-Baringo X.; Panigrahi A.; Larrosa I.; Casiraghi C. Insights into the exfoliation mechanism of pyrene-assisted liquid phase exfoliation of graphene from lateral size-thickness characterisation. Carbon 2022, 186, 550–559. 10.1016/j.carbon.2021.09.075.

[ref24] Just-Baringo X.; Shin Y.; Panigrahi A.; Zarattini M.; Nagyte V.; Zhao L.; Kostarelos K.; Casiraghi C.; Larrosa I. Palladium catalysed C–H arylation of pyrenes: access to a new class of exfoliating agents for water-based graphene dispersions. Chem. Sci. 2020, 11, 2472–2478. 10.1039/C9SC05101E. 34084412 PMC8157272

[ref25] Backes C.; Szydłowska B. M.; Harvey A.; Yuan S.; Vega-Mayoral V.; Davies B. R.; Zhao P.-l.; Hanlon D.; Santos E. J. G.; Katsnelson M. I.; et al. Production of Highly Monolayer Enriched Dispersions of Liquid-Exfoliated Nanosheets by Liquid Cascade Centrifugation. ACS Nano 2016, 10, 1589–1601. 10.1021/acsnano.5b07228. 26728793

[ref26] Backes C.; Campi D.; Szydlowska B. M.; Synnatschke K.; Ojala E.; Rashvand F.; Harvey A.; Griffin A.; Sofer Z.; Marzari N.; et al. Equipartition of Energy Defines the Size–Thickness Relationship in Liquid-Exfoliated Nanosheets. ACS Nano 2019, 13, 7050–7061. 10.1021/acsnano.9b02234. 31199123

[ref27] Torrisi F.; Hasan T.; Wu W.; Sun Z.; Lombardo A.; Kulmala T. S.; Hsieh G.-W.; Jung S.; Bonaccorso F.; Paul P. J.; et al. Inkjet-Printed Graphene Electronics. ACS Nano 2012, 6, 2992–3006. 10.1021/nn2044609. 22449258

[ref28] Li J.; Naiini M. M.; Vaziri S.; Lemme M. C.; Östling M. Inkjet Printing of MoS2. Adv. Funct. Mater. 2014, 24, 6524–6531. 10.1002/adfm.201400984.

[ref29] Reis N.; Derby B. Ink Jet Deposition of Ceramic Suspensions: Modeling and Experiments of Droplet Formation. MRS Online Proc. Library 2000, 625, 117 10.1557/PROC-625-117.

[ref30] Leng T.; Parvez K.; Pan K.; Ali J.; McManus D.; Novoselov K. S.; Casiraghi C.; Hu Z. Printed graphene/WS2 battery-free wireless photosensor on papers. 2D Mater. 2020, 7, 024004 10.1088/2053-1583/ab602f.

[ref31] McManus D.; Dal Santo A.; Selvasundaram P. B.; Krupke R.; LiBassi A.; Casiraghi C. Photocurrent study of all-printed photodetectors on paper made of different transition metal dichalcogenide nanosheets. Flex. Print. Electron. 2018, 3, 034005 10.1088/2058-8585/aaddb5.

[ref32] Peng Z.; Grillo A.; Pelella A.; Liu X.; Boyes M.; Xiao X.; Zhao M.; Wang J.; Hu Z.; Di Bartolomeo A.; et al. Fully printed memristors made with MoS2 and graphene water-based inks. Mater. Horiz. 2024, 11, 1344–1353. 10.1039/D3MH01224G. 38180062

[ref33] Kassem O.; Pimpolari L.; Dun C.; Polyushkin D. K.; Zarattini M.; Dimaggio E.; Chen L.; Basso G.; Parenti F.; Urban J. J.; et al. Water-based 2-dimensional anatase TiO2 inks for printed diodes and transistors. Nanoscale 2023, 15, 5689–5695. 10.1039/D2NR05786G. 36880645 PMC10035403

[ref34] Brunetti I.; Pimpolari L.; Conti S.; Worsley R.; Majee S.; Polyushkin D. K.; Paur M.; Dimaggio E.; Pennelli G.; Iannaccone G.; et al. Inkjet-printed low-dimensional materials-based complementary electronic circuits on paper. npj 2D Mater. Appl. 2021, 5, 85 10.1038/s41699-021-00266-5.

[ref35] Pimpolari L.; Calabrese G.; Conti S.; Worsley R.; Majee S.; Polyushkin D. K.; Paur M.; Casiraghi C.; Mueller T.; Iannaccone G.; Macucci M.; Fiori G.; et al. 1/f Noise Characterization of Bilayer MoS2 Field-Effect Transistors on Paper with Inkjet-Printed Contacts and hBN Dielectrics. Adv. Electron. Mater. 2021, 7, 2100283 10.1002/aelm.202100283.

[ref36] Conti S.; Pimpolari L.; Calabrese G.; Worsley R.; Majee S.; Polyushkin D. K.; Paur M.; Pace S.; Keum D. H.; Fabbri F.; et al. Low-voltage 2D materials-based printed field-effect transistors for integrated digital and analog electronics on paper. Nat. Commun. 2020, 11, 3566 10.1038/s41467-020-17297-z. 32678084 PMC7367304

[ref37] Lu S.; Cardenas J. A.; Worsley R.; Williams N. X.; Andrews J. B.; Casiraghi C.; Franklin A. D. Flexible, Print-in-Place 1D–2D Thin-Film Transistors Using Aerosol Jet Printing. ACS Nano 2019, 13, 11263–11272. 10.1021/acsnano.9b04337. 31578857

[ref38] Worsley R.; Pimpolari L.; McManus D.; Ge N.; Ionescu R.; Wittkopf J. A.; Alieva A.; Basso G.; Macucci M.; Iannaccone G.; et al. All-2D Material Inkjet-Printed Capacitors: Toward Fully Printed Integrated Circuits. ACS Nano 2019, 13, 54–60. 10.1021/acsnano.8b06464. 30452230

[ref39] Grillo A.; Peng Z.; Pelella A.; Di Bartolomeo A.; Casiraghi C. Etch and Print: Graphene-Based Diodes for Silicon Technology. ACS Nano 2023, 17, 1533–1540. 10.1021/acsnano.2c10684. PMC9878974 36475589

[ref40] Pimpolari L.; Brunetti I.; Sargeni R.; Pieri F.; Parvez K.; Macucci M.; Casiraghi C.; Fiori G. Fully Printed and Flexible Schottky Diodes Based on Carbon Nanomaterials Operating Up to 5 MHz. IEEE J. Flex. Electron. 2022, 1, 153–158. 10.1109/JFLEX.2022.3215928.

[ref41] Furlan de Oliveira R.; Montes-García V.; Ciesielski A.; Samorì P. Harnessing selectivity in chemical sensing via supramolecular interactions: from functionalization of nanomaterials to device applications. Mater. Horiz. 2021, 8, 2685–2708. 10.1039/D1MH01117K. 34605845

[ref42] Rohaizad N.; Mayorga-Martinez C. C.; Fojtů M.; Latiff N. M.; Pumera M. Two-dimensional materials in biomedical, biosensing and sensing applications. Chem. Soc. Rev. 2021, 50, 619–657. 10.1039/D0CS00150C. 33206730

[ref43] Song Y.; Lin B.; Tian T.; Xu X.; Wang W.; Ruan Q.; Guo J.; Zhu Z.; Yang C. Recent Progress in Microfluidics-Based Biosensing. Anal. Chem. 2019, 91, 388–404. 10.1021/acs.analchem.8b05007. 30412383

[ref44] Rivet C.; Lee H.; Hirsch A.; Hamilton S.; Lu H. Microfluidics for medical diagnostics and biosensors. Chem. Eng. Sci. 2011, 66, 1490–1507. 10.1016/j.ces.2010.08.015.

[ref45] Ongaro A. E.; Keraite I.; Liga A.; Conoscenti G.; Coles S.; Schulze H.; Bachmann T. T.; Parvez K.; Casiraghi C.; Howarth N.; et al. Laser Ablation of Poly(lactic acid) Sheets for the Rapid Prototyping of Sustainable, Single-Use, Disposable Medical Microcomponents. ACS Sustainable Chem. Eng. 2018, 6, 4899–4908. 10.1021/acssuschemeng.7b04348.

[ref46] Demuru S.; Huang C.-H.; Parvez K.; Worsley R.; Mattana G.; Piro B.; Noël V.; Casiraghi C.; Briand D. All-Inkjet-Printed Graphene-Gated Organic Electrochemical Transistors on Polymeric Foil as Highly Sensitive Enzymatic Biosensors. ACS Appl. Nano Mater. 2022, 5, 1664–1673. 10.1021/acsanm.1c04434.

[ref47] Casiraghi C.; Macucci M.; Parvez K.; Worsley R.; Shin Y.; Bronte F.; Borri C.; Paggi M.; Fiori G. Inkjet printed 2D-crystal based strain gauges on paper. Carbon 2018, 129, 462–467. 10.1016/j.carbon.2017.12.030.

[ref48] Conti S.; Nepa F.; Pascoli S. D.; Brunetti I.; Pimpolari L.; Song X.; Parvez K.; Lomeri H. J.; De Rossi F.; Lucarelli G.; et al. Hybrid Flexible NFC Sensor on Paper. IEEE J. Flex. Electron. 2023, 2, 4–10. 10.1109/JFLEX.2023.3238682.

[ref49] Lin H.; Buerki-Thurnherr T.; Kaur J.; Wick P.; Pelin M.; Tubaro A.; Carniel F. C.; Tretiach M.; Flahaut E.; Iglesias D.; et al. Environmental and Health Impacts of Graphene and Other Two-Dimensional Materials: A Graphene Flagship Perspective. ACS Nano 2024, 18, 6038–6094. 10.1021/acsnano.3c09699. 38350010 PMC10906101

[ref50] Andrews J. P. M.; Joshi S. S.; Tzolos E.; Syed M. B.; Cuthbert H.; Crica L. E.; Lozano N.; Okwelogu E.; Raftis J. B.; Bruce L.; et al. First-in-human controlled inhalation of thin graphene oxide nanosheets to study acute cardiorespiratory responses. Nat. Nanotechnol. 2024, 19, 705–714. 10.1038/s41565-023-01572-3. 38366225 PMC11106005

[ref51] Rodrigues A. F.; Newman L.; Lozano N.; Mukherjee S. P.; Fadeel B.; Bussy C.; Kostarelos K. A blueprint for the synthesis and characterisation of thin graphene oxide with controlled lateral dimensions for biomedicine. 2D Mater. 2018, 5, 035020 10.1088/2053-1583/aac05c.

[ref52] Munuera J.; Britnell L.; Santoro C.; Cuéllar-Franca R.; Casiraghi C. A review on sustainable production of graphene and related life cycle assessment. 2D Mater. 2022, 9, 012002 10.1088/2053-1583/ac3f23.

[ref53] Pinto A. M.; Moreira J. A.; Magalhães F. D.; Gonçalves I. C. Polymer surface adsorption as a strategy to improve the biocompatibility of graphene nanoplatelets. Colloids Surf. B: Biointerfaces 2016, 146, 818–824. 10.1016/j.colsurfb.2016.07.031. 27451370

[ref54] Fröhlich E. The role of surface charge in cellular uptake and cytotoxicity of medical nanoparticles. Int. J. Nanomed. 2012, 7, 5577–5591. 10.2147/IJN.S36111. PMC3493258 23144561

[ref55] Behzadi S.; Serpooshan V.; Tao W.; Hamaly M. A.; Alkawareek M. Y.; Dreaden E. C.; Brown D.; Alkilany A. M.; Farokhzad O. C.; Mahmoudi M. Cellular uptake of nanoparticles: journey inside the cell. Chem. Soc. Rev. 2017, 46, 4218–4244. 10.1039/C6CS00636A. 28585944 PMC5593313

[ref56] Chen Y.; Taufiq T.; Zeng N.; Lozano N.; Karakasidi A.; Church H.; Jovanovic A.; Jones S. A.; Panigrahi A.; Larrosa I.; et al. Defect-free graphene enhances enzyme delivery to fibroblasts derived from patients with lysosomal storage disorders. Nanoscale 2023, 15, 9348–9364. 10.1039/D2NR04971F. 37165691

[ref57] Peng G.; Keshavan S.; Delogu L.; Shin Y.; Casiraghi C.; Fadeel B. Two-Dimensional Transition Metal Dichalcogenides Trigger Trained Immunity in Human Macrophages through Epigenetic and Metabolic Pathways. Small 2022, 18, 2107816 10.1002/smll.202107816. 35434920

[ref58] Gazzi A.; Fusco L.; Orecchioni M.; Keshavan S.; Shin Y.; Grivel J.-C.; Rinchai D.; Ahmed E. I.; Elhanani O.; Furesi G.; et al. Immune profiling and tracking of two-dimensional transition metal dichalcogenides in cells and tissues. Nano Today 2024, 54, 102084 10.1016/j.nantod.2023.102084.

[ref59] Conti S.; Calabrese G.; Parvez K.; Pimpolari L.; Pieri F.; Iannaccone G.; Casiraghi C.; Fiori G. Printed transistors made of 2D material-based inks. Nat. Rev. Mater. 2023, 8, 651–667. 10.1038/s41578-023-00585-7.

[ref60] Rahman M.; Parvez K.; Fugallo G.; Dun C.; Read O.; Alieva A.; Urban J. J.; Lazzeri M.; Casiraghi C.; Pisana S. Anisotropic Thermal Conductivity of Inkjet-Printed 2D Crystal Films: Role of the Microstructure and Interfaces. Nanomaterials 2022, 12, 3861 10.3390/nano12213861. 36364636 PMC9654414

[ref61] Calabrese G.; Pimpolari L.; Conti S.; Mavier F.; Majee S.; Worsley R.; Wang Z.; Pieri F.; Basso G.; Pennelli G.; et al. Inkjet-printed graphene Hall mobility measurements and low-frequency noise characterization. Nanoscale 2020, 12, 6708–6716. 10.1039/C9NR09289G. 32186302

